# A pro-inflammatory and fibrous cap thinning transcriptome profile accompanies carotid plaque rupture leading to stroke

**DOI:** 10.1038/s41598-022-17546-9

**Published:** 2022-08-05

**Authors:** Hernan A. Bazan, Ashton J. Brooks, Kenny Vongbunyong, Christin Tee, Hunter F. Douglas, Natasha C. Klingenberg, T. Cooper Woods

**Affiliations:** 1 Section of Vascular/Endovascular Surgery, Department of Surgery, Ochsner Clinic Foundation, New Orleans, LA USA; 2 Faculty of Medicine, Ochsner Clinical School, The University of Queensland, New Orleans, LA USA; 3 Departments of Physiology and Medicine, Tulane University School of Medicine, 1430 Tulane Avenue, New Orleans, LA 70112 USA

**Keywords:** Vascular diseases, Cardiovascular genetics

## Abstract

Atherosclerotic plaque rupture is the etiology of ischemic stroke and myocardial infarction. The molecular mechanisms responsible for rupture remain unclear, in part, due to the lack of data from plaques at the time of rupture. Ribosome-depleted total RNA was sequenced from carotid plaques obtained from patients undergoing carotid endarterectomy with high-grade stenosis and either (1) a carotid-related ischemic cerebrovascular event within the previous 5 days ('recently ruptured,' n = 6) or (2) an absence of a cerebrovascular event ('asymptomatic,' n = 5). Principal component analysis confirmed plaque rupture was responsible for the greatest percentage of the variability between samples (23.2%), and recently ruptured plaques were enriched for transcripts associated with inflammation and extracellular matrix degradation. Hierarchical clustering achieved differentiation of the asymptomatic from the recently ruptured plaques. This analysis also found co-expression of transcripts for immunoglobulins and B lymphocyte function, matrix metalloproteinases, and interferon response genes. Examination of the differentially expressed genes supported the importance of inflammation and inhibition of proliferation and migration coupled with an increase in apoptosis. Thus, the transcriptome of recently ruptured plaques is enriched with transcripts associated with inflammation and fibrous cap thinning and support further examination of the role of B lymphocytes and interferons in atherosclerotic plaque rupture.

## Introduction

Atherosclerotic plaque rupture precipitates a thromboembolic event leading to ischemia. Clinical examples include acute neurological events, such as transient ischemic attacks and stroke, in the case of carotid arteries, and acute coronary syndromes in the case of coronary arteries^1,2^. The development of an atherosclerotic plaque is characterized by chronic inflammation, with the recruitment of monocyte-derived macrophages and T cells. These produce inflammatory mediators that enhance inflammation and promote vascular smooth muscle cells (VSMC) to proliferate and secrete collagen to form the fibrous cap^3^. In the late stage of atherosclerosis, thinning of the fibrous cap due to increased apoptosis of VSMCs and expression of matrix metalloproteinases create a rupture-prone thin cap fibroatheroma^4^.

Atherosclerotic plaques consist of a soft lipid-rich atheromatous core covered by a firm, collagen-rich fibrous cap which contains sclerotic tissue, VSMCs, and an intact endothelial lining^5^. Ruptured plaques have thinner overlying fibrous caps infiltrated by macrophages, T-lymphocytes, and B-lymphocytes^2^. The molecular mechanisms driving asymptomatic plaques to transition to those prone to rupture and cause atheroemboli remain unclear and require further study.

The features of the ruptured plaque have been primarily determined through histological analysis of tissue obtained from patients exhibiting symptoms within the past 180 days. While these data are quite valuable, our previous works suggest notable transient changes in RNA expression that occur with plaque rupture^6^. Here, we compared the transcriptomes of asymptomatic and recently (within 5 days) ruptured carotid plaques to determine molecular mechanisms active at the time of rupture.

## Results

### Patient population

Carotid plaques were obtained from 11 patients undergoing CEA, 5 were asymptomatic and 6 had an ischemic cerebrovascular event within the past 5 days (“recently ruptured”, Table 1). There was no significant difference in age, body mass index, lipid profile, serum creatinine, or estimated glomerular filtration rates between the two patient groups. Additionally, there similar rates of smoking, alcohol use and chronic kidney disease in both groups. All subjects were hypertensive while none were diabetic. Each group had similar levels of aspirin, statin, and clopidogrel use. The percent stenosis was similar between the two groups. The recently ruptured group consisted of 2 patients with crescendo transient ischemic attacks and 4 with NIH Stroke Severity Scores of 1, 3, 4, and 7. The time between the cerebrovascular event and the CEA of 3.5 ± 0.5 days.

**Table 1:** Patient characteristics.

	Total (n = 11)	Asymptomatic (n = 5)	Recently ruptured (n = 6)	*P*-value
**Characteristics**				
Age, y	67.2 ± 2.2	65.2 ± 3	68.8 ± 3.3	0.44
Body mass index, kg/m^2^	31.7 ± 1.8	30.8 ± 3	32.5 ± 2.3	0.65
History of smoking	8 (73%)	4 (80%)	4 (67%)	1.00
History of ethanol use	5 (45%)	3 (60%)	2 (33%)	0.57
Total cholesterol, mg/dL	145.5 ± 26	152.5 ± 11.5	140.8 ± 44.2	0.84
HDL, mg/dL	43.8 ± 3.2	46.3 ± 3.4	42.2 ± 4.9	0.57
LDL, mg/dL	102.9 ± 13.1	83.1 ± 11.7	116.1 ± 18.9	0.24
Triglycerides, mg/dL	141 ± 13	116 ± 11.2	157.7 ± 17.4	0.12
Serum Creatinine, mg/dL	1.2 ± 0.1	1.1 ± 0.1	1.3 ± 0.2	0.53
eGFR, mL/min/1.73m2	72.5 ± 7.2	76.7 ± 8.4	69.1 ± 11.7	0.12
Chronic kidney disease	3 (27%)	1 (20%)	2 (33%)	1.00
**Event to CEA time, days**			3.5 ± 0.5	
Percent Stenosis	78.1 ± 3.9	82.8 ± 4.2	74.2 ± 6.2	0.29
Aspirin use	6 (55%)	4 (80%)	2 (33%)	0.24
Statin use	7 (64%)	4 (80%)	3 (50%)	0.55
Clopidogrel use	2 (18%)	1 (20%)	1 (17%)	1.00

Data expressed as mean ± SEM.

### Principal component analysis of highly variable transcripts identifies a principal component associated with rupture

**Figure 1:**
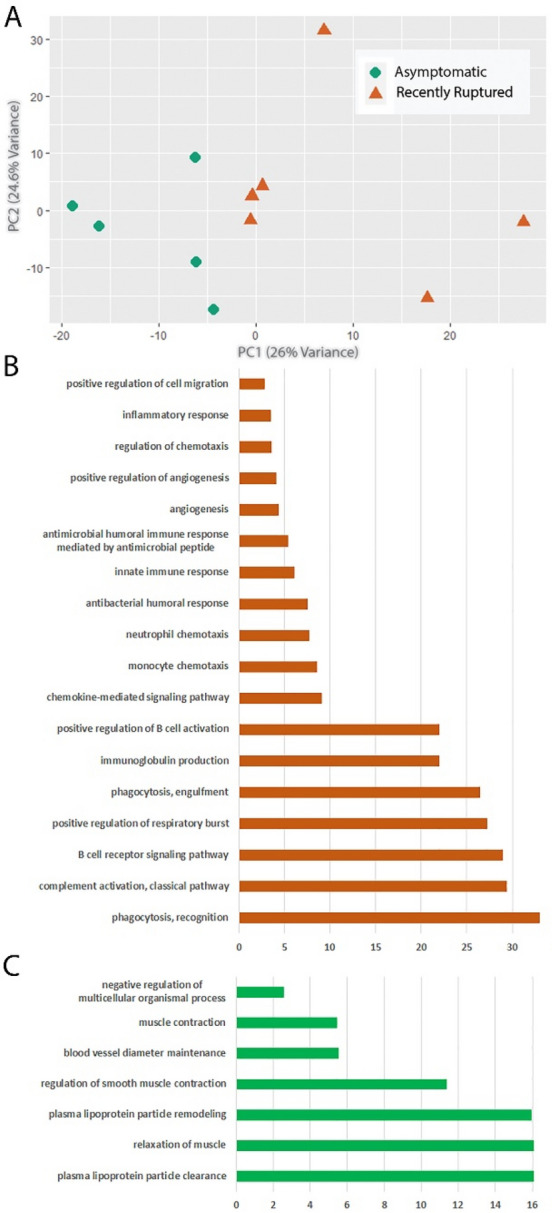
Principal component analysis (PCA) indicates plaque rupture is associated with alterations in the transcriptome. (**A**) PCA plot of the top 1750 most variable transcripts in asymptomatic and recently ruptured plaques. (**B**) Gene ontology of the transcripts associated with recently ruptured plaques. (**C**) Gene ontology of the transcripts associated with asymptomatic plaques.

PCA of the 1750 most variable transcripts found that the first principal component, which accounts for 26% of the total variability, represents the differences between the samples from asymptomatic versus recently ruptured groups (Fig. 1A). Gene ontology analysis of the transcripts with loadings associated with the recently symptomatic group, showed that this group was enriched for transcripts related to phagocytosis, B cell function, complement activation, inflammation and leukocyte chemotaxis (Fig. 1B). The loadings associated with the asymptomatic group were characterized by increased plasma lipoprotein particle clearance and remodeling and transcripts associated with regulation of muscle contraction (Fig. 1C).

### Hierarchical clustering of highly variable transcripts separates asymptomatic and recently symptomatic

**Figure 2:**
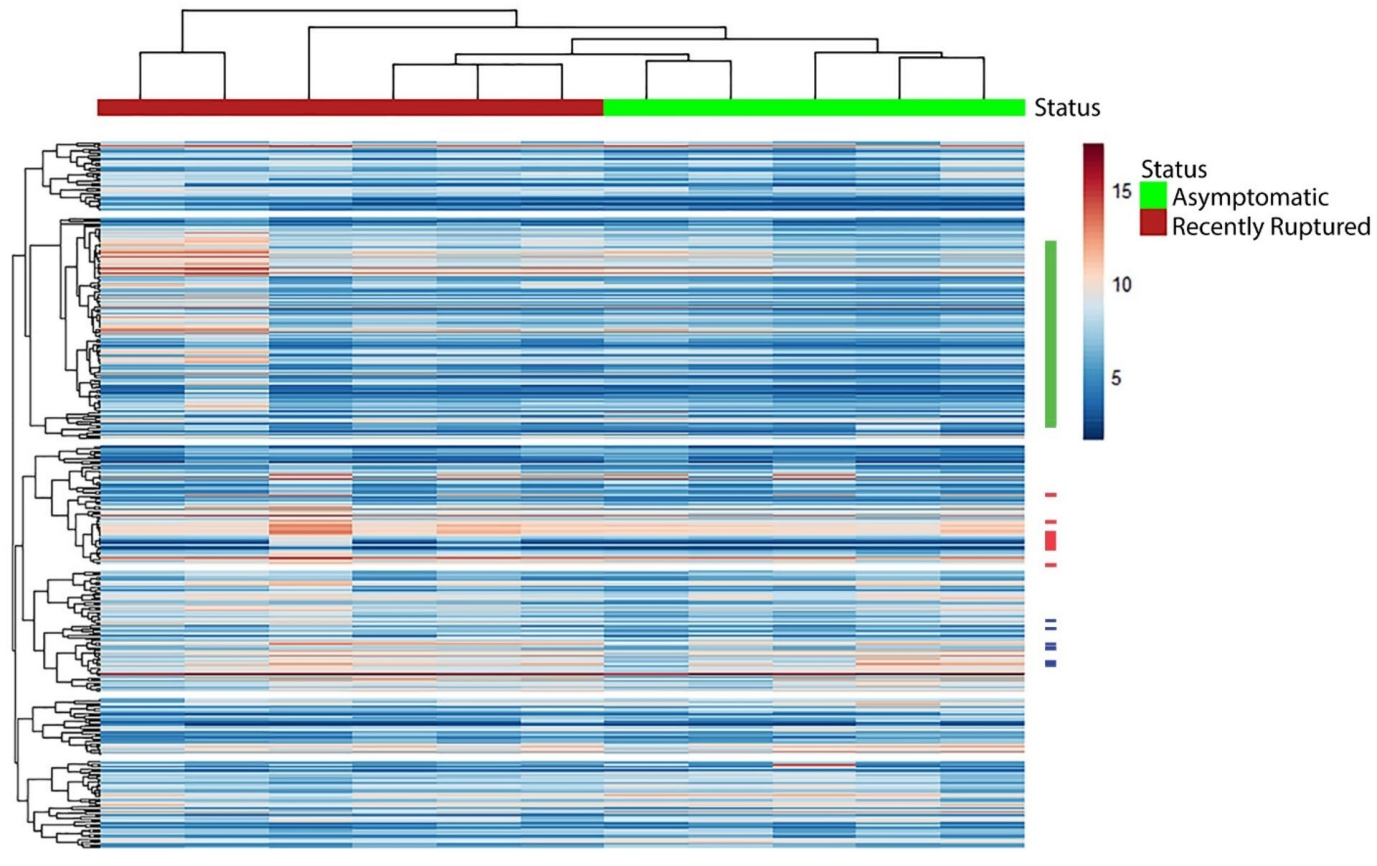
Hierarchical clustering identifies groups of co-expressed transcripts that distinguish recently ruptured plaques from asymptomatic plaques. Clustering of the top 400 transcripts in carotid plaques differentiates between asymptomatic and recently ruptured plaques. Bars adjacent to the cluster indicate locations of transcripts associated with B cell function (green), metalloproteinases (blue), and interferon responses (red). Supplementary Fig. 2 presents this data with the transcript names included.

To more directly examine the changes in gene expression that occur with plaque rupture, we performed hierarchical clustering of the transcripts in the carotid plaques (Fig. 2). Clustering a minimum of the 400 most variable transcripts samples achieved separation between the asymptomatic and recently ruptured plaque samples. Interestingly, while the asymptomatic samples clustered tightly together, two of the recently ruptured samples clustered away from all the other samples, suggesting greater variability in the recently ruptured plaque samples. We compared the individual demographic data for each patient with the patient clustering to determine if the patient characteristics might explain the groupings, but no clinical factor correlated with the sample dendrogram.

Overall, the heatmap does not present distinctive clusters of transcripts unique to asymptomatic or recently ruptured. Close inspection of the heatmap reveals a cluster of immunoglobulins and B cell function transcripts where expression is increased in the recently ruptured group. This was also true for clusters that include matrix metalloproteinases (e.g., *MMP1, MMP7, MMP8, MMP9, MMP12, MMP13, and MMP25)* and responses to interferon (e.g., *IFI6, IFI27, IFIT1*, *IFIT3, IFITM1, IFI44L, IRF7, ISG15, OAS2, OAS3,* and *OASL*) though the magnitude of the difference is less pronounced. Transcripts encoding inflammatory genes were scattered throughout the clusters, including numerous cytokines (e.g., *IL6, CXCL1, CXCL5, CXCL6, CXCL8, CXCL9,* and *CCL18*) exhibiting increases in the recently ruptured group.

### Differential gene expression underlying increased atherosclerotic plaque vulnerability and rupture

A total of 422 DEGs were identified from atherosclerotic plaque samples of recently ruptured compared to asymptomatic patients (p-adj < 0.05). Two hundred sixty-three transcripts were significantly increased, and 159 transcripts were significantly decreased (Supplementary Table 1). To better understand the biological processes behind the transition of stable to vulnerable atherosclerotic plaques, we explored significant transcripts of interest in more detail (Table 2).

**Table 2:** Differentially expressed genes of interest.

Gene symbol	Mean expression	Log_2_(fold change)	Standard error	Adjusted *P*-value
CD177	76	7.18	1.62	0.005
MZB1	192	2.82	0.83	0.039
SDC1	111	2.15	0.45	0.002
CD79A	72	2.14	0.63	0.039
RASA4B	107	2.09	0.48	0.007
XCR1	34	1.83	0.44	0.011
SH2D3C	125	1.43	0.35	0.011
SIK1	32	1.39	0.42	0.043
TP63	62	1.29	0.34	0.022
ZAP70	70	1.27	0.33	0.020
HMHA1	343	1.19	0.24	0.001
ARHGAP4	273	1.15	0.25	0.003
RASA4	535	1.12	0.31	0.028
ADAMTS4	246	1.11	0.30	0.028
SH3BP1	207	1.04	0.25	0.011
NPDC1	116	1.02	0.26	0.016
MIAT	287	1.02	0.29	0.036
DOK3	139	1.01	0.29	0.037
ZBTB17	116	1.00	0.25	0.012
TMSB15B	87	-1.03	0.31	0.041
MTRNR2L13	106	-3.24	0.97	0.043

Recently ruptured atherosclerotic plaques demonstrated increased expression of pro-inflammatory genes involved in the mobilization and recruitment of leukocytes to the vessel wall. *XCR1,* which encodes a chemokine receptor, and *CD177,* which mediates neutrophil activation, were increased in the recently ruptured group. The recently ruptured group also exhibited elevated levels of several transcripts associated with B cell function, including numerous immunoglobins and transcripts related to B cell proliferation (*MZB1)* and activation (*CD79A, SH2D3C,* and *ZAP70*).

Recently ruptured atherosclerotic plaque samples were also found to have a change in transcript expression that may relate to thinning of the fibrous cap through decreased VSMC proliferation and migration and increased apoptosis. *SDC1* and *SIK1* are associated with decreased VSMC proliferation and migration. Additionally, the recently ruptured plaques had increased expression of transcripts related to inhibition of cell proliferation (*NPDC1, DOK3,* and *ZBTB17*). Reduced expression of a pro-migration transcript (*TMSB15B*) was also observed in the recently ruptured samples. Several transcripts encode Ras GTPase-activating proteins (*RASA4, RASA4B, ARHGAP4, HMHA1,* and *SH3BP1*) that promote inactivation of Rac1 and cdc42, inhibiting proliferation and migration.

In addition to its anti-proliferative functions, *DOK3* is associated with increased apoptosis. Recently ruptured samples exhibited an increase in a pro-apoptotic transcript (*TP63)* and decreases in a transcript encoding an inhibitor of apoptosis (*MTRNR2L13*). Additionally, *ADAMTS4*, which encodes a proteinase able to degrade the extracellular matrix and has been identified as a potential marker of carotid plaque vulnerability, was upregulated in the recently ruptured samples^7^. The long non-coding RNA, *MIAT*, was significantly increased in the recently ruptured samples. This agrees with a previous report that *MIAT* is increased in ruptured compared to stable carotid plaques; however, *MIAT* promotes proliferation and inhibits apoptosis^8^.

## Discussion

The RNA-Seq dataset presented here demonstrates that recently ruptured plaques have a unique transcriptome that includes inflammatory and anti-proliferative features. The PCA analysis revealed that rupture status is the most significant contributor to the variability between samples. The hierarchical clustering demonstrated that the transcriptomes of the plaques share similarities according to plaque status as well. While expected, this common expression profile supports that the development of plaque vulnerability is an active process mediated by significant changes in gene expression.

Papaspyridonos et al*.* performed a similar analysis where CEA samples were collected and assessed macroscopically to stratify the samples into stable and unstable and a supervised analysis was used to identify DEGs between these groups. This study found 27 DEGs of interest that were confirmed by PCR^9^. Our study found Fold Changes in the same direction as in their study for 18 of these mRNAs. These similarities suggest that our dataset does include similar changes as seen in unstable plaques that have not necessarily ruptured.

Examination of the transcripts underlying the clustering of the samples revealed that many transcripts associated with the recently ruptured plaques play a role in inflammation, especially B cell function and type I interferon responses. B cells have been found in the fibrous caps of ruptured carotid plaques^2^. Analysis of the DEGs further supported an essential role for B cells in plaque rupture, with significant increases in transcripts associated with B cell function observed in the recently ruptured plaques. The role of B cells in atherosclerosis is dependent on subtype. B1a lymphocytes reduce the necrotic core size and thus are atheroprotective^10^. In contrast, the B2 subset promotes plaque development and vulnerability, potentially through secretion of tumor necrosis factor-α and increased apoptosis^11,12^. While many immunoglobulin transcripts were increased in the recently ruptured plaques, the lack of a significant increase in the transcript encoding IgM suggests that the B2 subset may predominate. Overall, these findings play an essential role for B2 cells in developing plaque vulnerability and rupture.

In addition to the B cells, our data also support enhanced inflammation. Type I interferons can be produced by multiple cell types in atherosclerotic plaques, including B cells, and promote foam cell formation and amplify inflammation. The hierarchical clustering indicated a set of co-expressed interferon response genes. *IFIT1* and *IFIT3* have been implicated in pro-inflammatory polarization of macrophages and decreased collagen deposition leading to plaque vulnerability^13,14^. Loss of *OAS2* and *OAS3* expression has been linked to reduced atherosclerotic plaque development in a mouse model of atherosclerosis^15^. Both the PCA and hierarchical clustering pointed to increased cytokine signaling. Overall, the data suggest an increase in inflammation associated with plaque rupture.

Our data also points to an increase in mechanisms that promote thinning of the fibrous cap. An enrichment of transcripts involved with matrix disassembly was associated with the recently ruptured group, and several matrix metalloproteinases were co-expressed in the hierarchical clustering. Similar to previous reports^7^, *ADAMTS4* was reduced in the recently ruptured group. Loss of *ADAMTS4* is associated with increased collagen content and overall plaque stability in a mouse model of atherosclerosis. The matrix metalloproteinases co-expressed in the hierarchical clustering target a broad spectrum of substrates, including collagens (*MMP1, MMP8*, and *MMP13*), gelatins (*MMP9*), elastins (*MMP12*), and fibronectin (*MMP7*)^16,17^. In a mouse model of plaque vulnerability, *MMP9* was associated with greater plaque stability, while *MMP7* attenuated VSMC content and *MMP12* promoted destabilization^18^. In contrast, overexpression of *MMP9* has been proposed to promote plaque destabilization^4^.

Several transcripts associated with decreased proliferation and migration were also upregulated in the recently ruptured plaques. Increased *SDC1* is associated with non-proliferating differentiated VSMCs, and *SIK1* is associated with decreased vascular remodeling and was increased in the ruptured samples^19,20^. There was also upregulation of several Ras GTPase-activating proteins. Members of this protein family have been implicated in the development of plaque vulnerability^21,22^. Additional transcripts suggest decreased proliferation and increased apoptosis occur at the time of rupture. Thinning of the fibrous cap occurs through decreased VSMC proliferation and increased VSMC apoptosis^23^.

It is important to note that this study measured RNA from whole artery homogenates. Thus, we cannot attribute an individual change in RNA expression to a specific cell type. Likewise, the observed changes in RNA expression may represent changes in the cellular composition of the plaque. Indeed, our data suggest an increase in B cell infiltration occurred in the recently ruptured plaques. Additionally, the samples were collected 2 to 5 days post-rupture and therefore include changes associated with the response to the rupture. We cannot dissect those changes present at the time of rupture from those that occur after rupture. Finally, additional studies are required to confirm that changes in protein expression accompany the changes in transcript abundance we report here. To our knowledge, however, this is the first study to perform RNA-Seq on carotid samples collected this close to the plaque rupture. Most studies comparing CEA samples include symptomatic plaques collected greater than 100 days post-rupture. Previously, we have reported that miR-221 and -222 are down-regulated in recently ruptured plaques but increase to levels similar to that found in asymptomatic plaques in 7 days^6^. Thus, these data likely include changes in RNA expression that have previously been missed. Previous histologic analyses of carotid plaque rupture found "scarce" B cell infiltration^22^. Our data support a robust role for B cells in plaque rupture.

## Conclusion

Overall, these data present a picture of significant changes in the inflammation within the plaque associated with rupture, including a vital role for B cells. Additionally, an increase in a broad spectrum of matrix metalloproteinases coupled with the loss of VSMCs underlies thinning of the fibrous cap. Future studies on changes in RNA expression that can be localized to specific regions or cells within the plaque will further enhance our understanding of the molecular mechanisms driving plaque rupture.

## Materials and methods

### Sample collection

Carotid plaque specimens were obtained from a total of 11 patients who underwent carotid endarterectomy (CEA) in the Section of Vascular/Endovascular Surgery in the Department of Surgery at the Ochsner Clinic in New Orleans, LA. Samples obtained from patients undergoing CEA were stratified into two distinct clinical phenotypes: asymptomatic and recently ruptured. The asymptomatic group included patients undergoing CEA without a previous neurologic event but high-grade carotid stenosis (n = 5). The recently ruptured group (n = 6) consists of patients undergoing CEA within 5 days of an acute neurologic event (transient ischemic attack or acute stroke). The samples were immediately snap-frozen and stored in liquid nitrogen until processing for RNA isolation.

All procedures in this study were performed in accordance with the ethical standards of the 1964 Helsinki Declaration. This study was approved by the Ochsner Health institutional review board (IRB protocol # 2010.130B). All of the participants gave written informed consent for their participation in the study.

### RNA isolation

Carotid plaques were sectioned into 6 transverse segments of ~ 1 cm in length and macroscopic examination was performed to determine the sections with greatest plaque content. Sections containing a complicated lesion were homogenized in TRIzol Reagent (ThermoFisher) using a rotor stator homogenizer and total RNA was isolated according to the manufacturer's instructions. Total RNA was further purified using the miRNeasy Mini Kit (Qiagen).

### RNA-sequencing

The University Wisconsin Biotechnology Center performed library preparation and sequencing. Ribosomal RNA was reduced using the RiboZero assay (EpiCentre-Illumina), and an RNA-Seq library was constructed with the stranded TruSeq RNA kit (Illumina). Acceptable quality of each RNA sample and DNA library was confirmed using the 2100 Bioanalyzer system. Using a HiSeq2000 (Illumina), 1 × 100 bp sequencing was performed at average depth of 42 M reads per sample. The Tulane Next Generation Sequence Analysis Core aligned RNA-Seq reads to the human genome (hg19 assembly) using Novoalign (Novocraft), and splice junctions were identified using TopHat^24^. Coverage files and FPKM values were generated using SAMMate, and coverage was visualized on the Integrated Genome Viewer^25,26^.

Transcript abundance estimates were imported using *tximport* and package and principal component analysis (PCA), hierarchical clustering, and identification of differentially expressed genes (DEGs) were performed in an unsupervised manner using the *DESeq2* package^27,28^. DEGs were defined as those transcripts with a p-adjusted value < 0.05 with a fold change > 2. Gene ontology was performed using the PANTHER classification system with a minimum enrichment value of 1 and false discovery rate < 0.012^29^. The datasets generated and/or analysed during the current study are available in the [NCBI GEO (Gene Expression Omnibus)] repository, [PERSISTENT WEB INK OR GSE198600 TO DATASETS].

### Validation of RNA-Seq dataset with Droplet Digital PCR

The results of the RNA sequencing were validated by comparing the log_2_(Fold Change) obtained from the RNA-Seq analysis to that measured by polymerase chain reaction (PCR) of mRNAs selected randomly from a preliminary list of DEGs in an additional set of asymptomatic and recently ruptured samples (Supplementary Fig. 1), n = 5–16/group). The appropriate Quantitect Primer Assays (Qiagen) was paired with QX200 ddPCR EvaGreen PCR supermix (BioRad) to perform a one-step RT-PCR. QX200 droplet reader (BioRad) paired with QuantaSoft Analysis Pro v1.0 software (BioRad) for direct quantification of individual target molecules from each sample.

## Supplementary Information

Supplementary Information.

## Data Availability

The datasets generated during and analyzed during the current study are available in the Gene Expression Omnibus repository under the accession number GSE198600.

## Abbreviations

CEA: Carotid endarterectomy
DEG: Differentially expressed gene
PCA: Principal component analysis
VSMC: Vascular smooth muscle cell
