# Supplementary material for: A pro-inflammatory and fibrous cap thinning transcriptome profile accompanies carotid plaque rupture leading to stroke
Source: Sci Rep. 2022 Aug 5;12:13499. doi: 10.1038/s41598-022-17546-9 (PMC9356141; doi:10.1038/s41598-022-17546-9)
Supplement: Supplementary file 1 — Supplementary Information. [file 41598_2022_17546_MOESM1_ESM.pdf]

Supplemental Table 1

Gene Symbol	Mean Expression	Log_2(FoldChange)	Standard Error	Adjusted P-value
CD177	76	7.2	1.6	0.005
LBP	19	5.8	1.4	0.013
MTND1P23	193	5.2	1.4	0.028
IGKV1-9	599	4.0	1.0	0.021
IGHG4	4661	3.6	1.0	0.037
IGHV4-59	981	3.6	0.9	0.021
NUTM2A	12	3.5	0.7	0.002
RP11-731F5.2	108	3.5	1.0	0.035
IGHV4-34	136	3.4	0.7	0.003
IGKV3-15	1213	3.3	0.9	0.019
IGHV1OR15-4	14	3.3	1.0	0.038
RP3-410C9.1	24	3.3	0.8	0.011
IGHV1-58	34	3.3	0.8	0.013
IGHV4-55	86	3.2	0.9	0.028
IGHV1-46	228	3.2	0.8	0.022
IGHG3	3121	3.2	0.9	0.038
IGHV5-51	553	3.1	0.9	0.039
IGHV4-61	424	3.0	0.8	0.021
IGKV1-17	310	3.0	0.8	0.020
IGHA2	386	3.0	0.7	0.010
IGHV3-49	213	3.0	0.6	0.000
IGHV3-33	538	2.9	0.9	0.050
IGHV4-28	73	2.9	0.6	0.003
IGHV3-64	105	2.9	0.9	0.043
IGKV1-8	3964	2.8	0.8	0.028
MZB1	192	2.8	0.8	0.039
IGLV3-9	81	2.7	0.8	0.038
IGKV1-5	1462	2.7	0.8	0.032
IGKV3OR2-268	21	2.7	0.8	0.036
PTGDS	259	2.5	0.3	0.000
IGLV2-23	552	2.5	0.6	0.019
VMO1	85	2.4	0.5	0.002
IGLV3-27	25	2.4	0.6	0.020
GJB2	18	2.4	0.6	0.009
RN7SL184P	82	2.3	0.5	0.003
SLC30A8	15	2.3	0.6	0.016
SDC1	111	2.1	0.4	0.002
CD79A	72	2.1	0.6	0.039
IGHA1	2940	2.1	0.6	0.037
OPLAH	14	2.1	0.6	0.046

RASA4B	107	2.1	0.5	0.007
FGFR3	17	1.9	0.5	0.020
RMRP	19929	1.9	0.5	0.016
XCR1	34	1.8	0.4	0.011
LAMP5	31	1.8	0.4	0.011
ALPL	74	1.8	0.5	0.039
NLGN4X	220	1.8	0.4	0.010
ODF3B	42	1.7	0.5	0.028
RP1-29C18.8	27	1.7	0.5	0.028
C7	944	1.7	0.5	0.028
SHANK3	134	1.6	0.4	0.006
SPAG4	28	1.6	0.4	0.020
CNDP1	19	1.6	0.4	0.020
FCRL5	303	1.6	0.5	0.043
HSH2D	78	1.5	0.4	0.015
GNB1L	16	1.5	0.4	0.013
SYT7	17	1.5	0.5	0.041
FAM69B	24	1.5	0.5	0.039
TSPAN11	108	1.5	0.3	0.000
RN7SL44P	63	1.5	0.4	0.037
AC005757.7	12	1.5	0.4	0.025
GRM7	19	1.5	0.4	0.039
CKB	60	1.5	0.4	0.046
SCAND1	35	1.5	0.4	0.041
SPATA2L	24	1.5	0.4	0.010
AGRN	199	1.5	0.4	0.032
BMPER	110	1.5	0.4	0.022
LIPG	169	1.5	0.3	0.005
ATP5D	74	1.4	0.4	0.022
SH2D3C	125	1.4	0.3	0.011
SLC9A3R2	149	1.4	0.4	0.022
FAM221B	20	1.4	0.4	0.029
RN7SL524P	13	1.4	0.4	0.046
AC006538.1	16	1.4	0.4	0.035
LAX1	135	1.4	0.4	0.019
SIK1	32	1.4	0.4	0.043
RN7SL823P	47	1.4	0.4	0.041
PRAM1	48	1.4	0.4	0.029
RAMP3	132	1.4	0.4	0.046
KIF6	47	1.4	0.3	0.001
ISG20	121	1.4	0.4	0.045
FAAH2	17	1.4	0.4	0.038
MED16	81	1.3	0.4	0.033

DAGLA	55	1.3	0.4	0.028
RN7SL635P	96	1.3	0.4	0.033
GGT5	183	1.3	0.3	0.003
ZBTB46	110	1.3	0.2	0.000
AC017006.3	15	1.3	0.4	0.028
P2RX1	44	1.3	0.3	0.002
RAVER1	82	1.3	0.4	0.039
TP63	62	1.3	0.3	0.022
FER1L4	74	1.3	0.4	0.032
ZAP70	70	1.3	0.3	0.020
HYAL2	474	1.3	0.3	0.025
C22orf34	44	1.3	0.3	0.019
APOBR	118	1.3	0.4	0.034
ZNF467	27	1.2	0.4	0.042
CARD9	43	1.2	0.3	0.021
SMPD3	21	1.2	0.3	0.010
HIST1H4J	728	1.2	0.4	0.046
TRABD	87	1.2	0.3	0.003
L1TD1	23	1.2	0.4	0.038
GPR114	20	1.2	0.3	0.026
SIGIRR	70	1.2	0.3	0.014
ZNF414	36	1.2	0.3	0.031
LIF	38	1.2	0.4	0.046
C19orf24	32	1.2	0.3	0.037
HMHA1	343	1.2	0.2	0.001
CCDC88B	147	1.2	0.3	0.017
C14orf80	37	1.2	0.3	0.025
HDAC10	55	1.2	0.3	0.010
ITGB4	167	1.2	0.3	0.021
RP11-551L14.1	18	1.2	0.4	0.047
ORAI1	82	1.2	0.3	0.011
TCIRG1	420	1.2	0.3	0.029
POLD1	77	1.2	0.2	0.002
ARHGAP4	273	1.2	0.2	0.003
GFRA2	59	1.1	0.3	0.034
LTB	30	1.1	0.3	0.042
ADAMTSL2	50	1.1	0.3	0.015
KB-1980E6.2	21	1.1	0.3	0.037
SIRT6	45	1.1	0.3	0.041
RASA4	535	1.1	0.3	0.028
PATL2	47	1.1	0.3	0.039
SPPL2B	74	1.1	0.3	0.036
FCHO1	54	1.1	0.2	0.000

ADAMTS4	246	1.1	0.3	0.028
RP11-78F17.1	30	1.1	0.3	0.045
GALK1	51	1.1	0.3	0.020
FES	213	1.1	0.2	0.002
SHISA2	23	1.1	0.3	0.038
LTB4R2	21	1.1	0.3	0.041
CEP131	34	1.1	0.3	0.045
BOP1	68	1.1	0.3	0.010
LLGL2	47	1.1	0.3	0.029
ABCA7	86	1.1	0.3	0.029
FAM195A	42	1.1	0.3	0.025
NOC4L	25	1.1	0.3	0.034
IKBKE	143	1.1	0.3	0.043
PASK	78	1.1	0.2	0.001
HIST1H1E	731	1.0	0.3	0.028
ADCY4	161	1.0	0.2	0.001
SH3BP1	207	1.0	0.3	0.011
ATP2A3	323	1.0	0.3	0.019
RLTPR	22	1.0	0.3	0.029
NDRG4	101	1.0	0.3	0.036
FCRL2	30	1.0	0.3	0.046
NPDC1	116	1.0	0.3	0.016
MIAT	287	1.0	0.3	0.036
WDR24	38	1.0	0.3	0.046
DOK3	139	1.0	0.3	0.037
ESR2	21	1.0	0.3	0.041
CLNK	34	1.0	0.3	0.043
ZBTB17	116	1.0	0.2	0.012
H1FX	176	1.0	0.3	0.028
LRCH4	186	1.0	0.2	0.006
EXOSC5	40	1.0	0.3	0.020
CORO1A	539	1.0	0.3	0.041
CACNA1D	36	1.0	0.3	0.042
CRTAC1	1668	1.0	0.3	0.023
SNORD118	39	1.0	0.3	0.038
ACAP1	161	1.0	0.2	0.000
CSK	469	1.0	0.3	0.039
KMT2B	261	1.0	0.2	0.009
ADAMTSL4	198	1.0	0.3	0.037
RP11-902B17.1	36	1.0	0.3	0.037
TP53I13	82	1.0	0.3	0.024
MYH3	90	1.0	0.2	0.020
C15orf39	233	0.9	0.2	0.015

TICAM1	69	0.9	0.2	0.014
NCF1B	72	0.9	0.3	0.031
TONSL	48	0.9	0.3	0.041
RPLP0P2	49	0.9	0.3	0.042
SH3BP2	1145	0.9	0.2	0.011
AC007308.6	30	0.9	0.3	0.049
SELO	55	0.9	0.2	0.028
DCN	10607	0.9	0.3	0.043
FHDC1	47	0.9	0.2	0.020
FMNL1	627	0.9	0.2	0.016
ABHD17A	111	0.9	0.3	0.047
PSD4	570	0.9	0.2	0.010
MFSD10	87	0.9	0.3	0.046
GPR132	120	0.9	0.2	0.033
SPN	354	0.9	0.2	0.028
RNF44	223	0.9	0.1	0.000
CLSTN3	187	0.9	0.2	0.030
KLF16	80	0.9	0.2	0.027
E4F1	80	0.8	0.2	0.038
LYL1	74	0.8	0.3	0.042
DGKZ	422	0.8	0.2	0.039
C10orf54	677	0.8	0.3	0.046
RN7SL144P	235	0.8	0.3	0.047
EPN1	254	0.8	0.2	0.039
SH3TC1	254	0.8	0.2	0.037
ST3GAL2	456	0.8	0.2	0.010
KIFC3	286	0.8	0.2	0.039
WDR81	314	0.8	0.2	0.038
MBD3	154	0.8	0.2	0.039
C17orf70	97	0.8	0.2	0.029
TECPR1	252	0.8	0.2	0.019
WAS	295	0.8	0.2	0.041
IKBKG	117	0.8	0.2	0.034
UNC13D	220	0.8	0.2	0.039
UBXN11	123	0.8	0.2	0.038
PSTPIP1	129	0.8	0.2	0.039
ATG16L2	308	0.8	0.2	0.022
CSF1R	3391	0.8	0.2	0.029
FUOM	70	0.8	0.2	0.020
PHRF1	213	0.8	0.2	0.010
ARHGAP27	316	0.8	0.2	0.039
SEC31B	135	0.8	0.2	0.016
ABCA6	740	0.8	0.2	0.020

CTDP1	159	0.8	0.2	0.017
ZNF219	73	0.8	0.2	0.045
FURIN	513	0.8	0.2	0.038
WDR4	49	0.7	0.2	0.039
RASAL3	125	0.7	0.2	0.041
RHOF	153	0.7	0.2	0.025
RASGRP4	118	0.7	0.2	0.044
BMP1	298	0.7	0.2	0.019
PIEZO1	438	0.7	0.2	0.014
LAT	123	0.7	0.2	0.010
KIF21B	203	0.7	0.2	0.049
RHBDD3	55	0.7	0.2	0.035
CORO7	264	0.7	0.2	0.045
MYO15B	244	0.7	0.2	0.035
ARID3A	148	0.7	0.2	0.044
ZNF646	267	0.7	0.2	0.038
RPH3AL	50	0.7	0.2	0.048
PTOV1	259	0.7	0.2	0.037
NCKAP5L	280	0.7	0.2	0.028
KIAA1217	460	0.7	0.2	0.041
MAP4K1	151	0.7	0.2	0.011
LLGL1	183	0.7	0.2	0.020
DDX11	190	0.7	0.2	0.041
MBD6	190	0.7	0.2	0.032
KDM4B	267	0.6	0.2	0.041
SEMA4D	549	0.6	0.2	0.044
RXRA	695	0.6	0.2	0.039
MAP3K14	226	0.6	0.2	0.035
PARVG	646	0.6	0.2	0.039
C1orf186	65	0.6	0.2	0.028
ANKRD13D	245	0.6	0.2	0.042
PTK2B	736	0.6	0.2	0.026
DAGLB	264	0.6	0.1	0.003
ORAI2	462	0.6	0.1	0.005
DENND4B	437	0.6	0.2	0.030
SPG7	508	0.6	0.2	0.037
CDK10	226	0.6	0.2	0.037
TRMT1	106	0.6	0.2	0.036
UCKL1	122	0.5	0.1	0.022
RNF31	238	0.5	0.1	0.020
TRIM8	754	0.5	0.2	0.039
CTD-3074O7.12	102	0.5	0.2	0.033
MSH5	141	0.5	0.2	0.042

GIGYF1	339	0.5	0.2	0.039
AMPD2	356	0.5	0.1	0.022
MZF1	117	0.5	0.2	0.041
APBB3	144	0.5	0.1	0.047
NOMO2	1667	0.5	0.1	0.038
NLRP1	960	0.4	0.1	0.034
IKBKB	969	0.4	0.1	0.029
PPIL2	406	0.4	0.1	0.034
DDX6	6598	-0.2	0.1	0.042
C16orf72	5939	-0.3	0.1	0.045
WAC	3282	-0.3	0.1	0.019
TTC37	3928	-0.3	0.1	0.046
SKIV2L2	1829	-0.3	0.1	0.020
DIAPH2	2389	-0.3	0.1	0.009
CTNNA1	5876	-0.3	0.1	0.034
TP53BP2	1387	-0.3	0.1	0.038
CD46	5555	-0.3	0.1	0.038
RTF1	1030	-0.3	0.1	0.039
LONP2	2413	-0.3	0.1	0.039
PDHA1	596	-0.3	0.1	0.047
MTR	2724	-0.3	0.1	0.038
MUT	779	-0.3	0.1	0.018
RAB3GAP1	2473	-0.3	0.1	0.046
ZNF654	909	-0.3	0.1	0.048
TMEM245	2785	-0.3	0.1	0.028
PTGES3	4460	-0.3	0.1	0.038
DYNC1I2	2773	-0.3	0.1	0.046
HIVEP2	2160	-0.3	0.1	0.030
PIP4K2C	519	-0.3	0.1	0.023
TOMM70A	1697	-0.3	0.1	0.047
SOCS5	1539	-0.3	0.1	0.045
PMPCB	989	-0.3	0.1	0.043
CCDC6	2970	-0.3	0.1	0.033
ATG14	722	-0.3	0.1	0.016
LRPPRC	2642	-0.3	0.1	0.048
VPS45	870	-0.4	0.1	0.039
VKORC1L1	1075	-0.4	0.1	0.031
SEPT11	4289	-0.4	0.1	0.034
DTWD1	804	-0.4	0.1	0.045
LTN1	2312	-0.4	0.1	0.028
OPTN	2500	-0.4	0.1	0.041
ANXA11	2975	-0.4	0.1	0.037
ZBTB41	1816	-0.4	0.1	0.039

ARHGAP12	1937	-0.4	0.1	0.039
KIAA0232	2021	-0.4	0.1	0.044
KIFAP3	1246	-0.4	0.1	0.035
APOOL	844	-0.4	0.1	0.042
DDX1	1901	-0.4	0.1	0.028
PLEKHA1	2154	-0.4	0.1	0.029
LYRM4	433	-0.4	0.1	0.046
MSH6	1014	-0.4	0.1	0.039
ARMC9	533	-0.4	0.1	0.046
MMGT1	1588	-0.4	0.1	0.033
SLC39A6	1712	-0.4	0.1	0.044
VEZT	1834	-0.4	0.1	0.019
CYB5R1	825	-0.4	0.1	0.012
PJA2	8874	-0.4	0.1	0.016
IARS2	1902	-0.4	0.1	0.045
ESYT2	5953	-0.4	0.1	0.041
KCTD2	615	-0.4	0.1	0.043
GINM1	1503	-0.4	0.1	0.040
HIBADH	581	-0.4	0.1	0.042
RAB22A	2324	-0.4	0.1	0.039
MCU	628	-0.4	0.1	0.038
COPS4	799	-0.4	0.1	0.039
RRM1	804	-0.4	0.1	0.039
HCFC2	1301	-0.4	0.1	0.016
ANKMY2	475	-0.4	0.1	0.020
ZNF569	288	-0.4	0.1	0.038
SCRN1	2403	-0.4	0.1	0.029
ATP6V1E1	1875	-0.4	0.1	0.044
DDHD2	1309	-0.4	0.1	0.025
FSTL1	16252	-0.4	0.1	0.007
PAPD5	656	-0.4	0.1	0.039
SPIN1	3015	-0.4	0.1	0.029
LMBRD2	1411	-0.4	0.1	0.023
VDAC2	1809	-0.4	0.1	0.040
ANKRD50	2010	-0.5	0.1	0.039
SLC35F5	3362	-0.5	0.1	0.003
KIF13A	3298	-0.5	0.1	0.041
RSU1	2560	-0.5	0.1	0.037
TMBIM1	4308	-0.5	0.1	0.028
IPP	296	-0.5	0.1	0.043
HSDL2	1501	-0.5	0.1	0.042
ZNF232	115	-0.5	0.1	0.038
TRAK2	4410	-0.5	0.1	0.039

HIPK3	10020	-0.5	0.1	0.034
KDELC2	2123	-0.5	0.1	0.038
SETD8	694	-0.5	0.1	0.038
GTDC1	957	-0.5	0.1	0.039
RAB6A	4236	-0.5	0.1	0.038
AK3	1626	-0.5	0.1	0.029
ARHGAP5	5850	-0.5	0.1	0.039
ARMCX6	371	-0.5	0.1	0.040
BTBD10	978	-0.5	0.1	0.036
ANO6	6337	-0.5	0.1	0.028
SEC22A	508	-0.5	0.2	0.046
CCND2	5655	-0.5	0.2	0.045
ACAT1	824	-0.5	0.1	0.010
CCND1	4017	-0.5	0.1	0.020
FAM63B	3023	-0.5	0.1	0.019
C12orf29	589	-0.5	0.1	0.013
SERINC1	12223	-0.5	0.1	0.017
GBE1	1890	-0.5	0.1	0.020
HSDL1	452	-0.5	0.1	0.016
RNF14	1671	-0.5	0.2	0.037
PCGF5	10647	-0.5	0.2	0.043
ATL3	11471	-0.5	0.1	0.020
MGST3	1639	-0.5	0.2	0.035
PGRMC1	1723	-0.5	0.1	0.010
FAM213A	918	-0.5	0.1	0.007
ITGB1	37950	-0.5	0.2	0.041
ENDOD1	2720	-0.5	0.1	0.020
PRKAB2	1051	-0.6	0.1	0.000
ATL1	285	-0.6	0.2	0.037
CLIC4	22740	-0.6	0.1	0.022
AVEN	350	-0.6	0.1	0.022
ARMCX1	822	-0.6	0.2	0.047
HTRA1	7909	-0.6	0.2	0.036
NIPSNAP3A	953	-0.6	0.2	0.045
PRMT5	545	-0.6	0.2	0.039
ADH5	3746	-0.6	0.2	0.020
ACTR10	1214	-0.6	0.1	0.016
RASSF3	3875	-0.6	0.2	0.038
NEK7	6445	-0.6	0.2	0.037
METTL18	183	-0.6	0.2	0.040
GNG12	6090	-0.6	0.2	0.020
FOXL1	283	-0.6	0.2	0.031
PRSS23	6124	-0.6	0.2	0.046

BMPR1A	2601	-0.6	0.2	0.046
TGFB2	729	-0.6	0.2	0.041
TUBA1B	14288	-0.7	0.1	0.002
HAPLN1	1071	-0.7	0.2	0.050
SLC41A2	1843	-0.7	0.2	0.030
WHAMMP3	397	-0.7	0.2	0.003
ZNF239	68	-0.7	0.2	0.041
RP11-400K9.4	407	-0.7	0.2	0.040
DSCC1	80	-0.8	0.2	0.020
SGCE	651	-0.8	0.2	0.022
PNMA2	776	-0.8	0.2	0.000
ERP27	88	-0.8	0.2	0.038
XG	239	-0.8	0.2	0.038
BVES	293	-0.8	0.2	0.019
GLDN	1044	-0.8	0.2	0.020
ADSSL1	202	-0.9	0.3	0.030
BMPR1APS2	62	-0.9	0.3	0.023
GTF2H2B	212	-1.0	0.3	0.033
KIAA0408	97	-1.0	0.2	0.014
AC093616.4	105	-1.0	0.3	0.020
GRHL2	74	-1.0	0.2	0.000
CTD-3092A11.1	219	-1.0	0.2	0.002
CATSPERB	91	-1.0	0.3	0.046
CKMT2	88	-1.0	0.3	0.039
TMSB15B	87	-1.0	0.3	0.041
PGBD3	127	-1.1	0.3	0.037
CRYZ	920	-1.1	0.2	0.000
HTR2B	424	-1.2	0.3	0.037
AURKC	14	-1.2	0.4	0.042
ADORA3	787	-1.4	0.3	0.013
AJAP1	40	-1.6	0.4	0.025
RP11-65I12.1	162	-1.7	0.4	0.019
PAX8-AS1	364	-1.8	0.5	0.039
RP11-632K20.2	102	-1.8	0.4	0.001
LRRC39	84	-1.9	0.4	0.001
INTS4L1	17	-2.3	0.7	0.044
RPL3P4	94	-3.1	0.8	0.019
MTRNR2L13	106	-3.2	1.0	0.043

Supplemental Figure 1. Comparison of Log_2(Fold Change) values from the RNA-Seq analysis and PCR measurement of randomly selected transcripts. Selected transcripts (n = 25) identified in the DEGs were measured in additional CEA samples using droplet digital PCR and the log_2 (Fold Change) was compared to that observed in the DEG analysis. A minimum of 5 samples / group were used with some targets being measured in up to 16 samples / group.

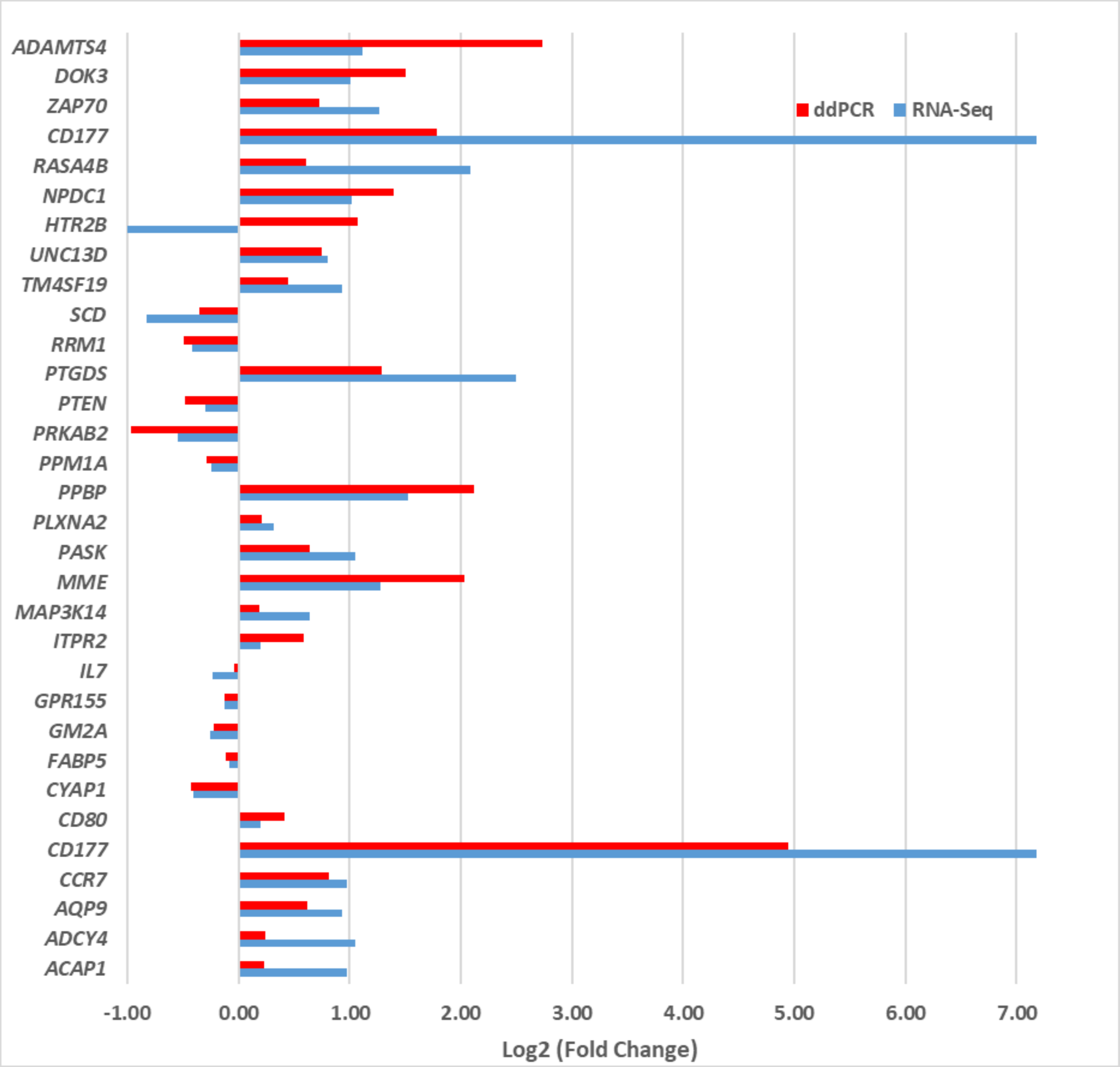

Supplemental Figure 2. Hierarchical clustering identifies groups of co-expressed transcripts that distinguish recently ruptured plaques from asymptomatic plaques. Clustering of the top 400 transcripts in carotid plaques differentiates between asymptomatic and recently ruptured plaques. Bars adjacent to the cluster indicate locations of transcripts associated with B-cell function (green), metalloproteinases (blue), and interferon responses (red). Note: this is the same figure as Figure 1 in the manuscript, but it includes the transcript names.

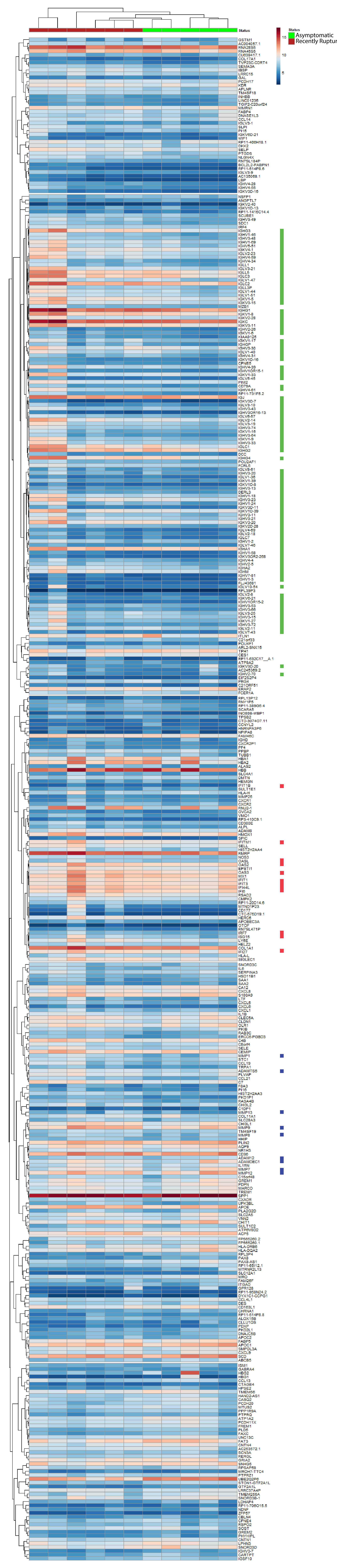

Status
Status
Asymptomatic
Recently Ruptur
15
10
5
